# *In Vitro* Enzymatic Studies Reveal pH and Temperature Sensitive Properties of the CLIC Proteins

**DOI:** 10.3390/biom13091394

**Published:** 2023-09-15

**Authors:** Amani Alghalayini, Khondker Rufaka Hossain, Saba Moghaddasi, Daniel R. Turkewitz, Claudia D’Amario, Michael Wallach, Stella M. Valenzuela

**Affiliations:** 1 School of Life Sciences, University of Technology Sydney, Sydney, NSW 2007, Australia; amani.alghalayini@uts.edu.au (A.A.); khondker.hossain@uts.edu.au (K.R.H.); saba.moghaddasi@alumni.uts.edu.au (S.M.); daniel.r.turkewitz@alumni.uts.edu.au (D.R.T.); claudia.damario@alumni.uts.edu.au (C.D.); michael.wallach@uts.edu.au (M.W.); 2 ARC Research Hub for Integrated Device for End-User Analysis at Low-Levels (IDEAL), Faculty of Science, University of Technology Sydney, Sydney, NSW 2007, Australia

**Keywords:** CLIC proteins, oxidoreductase enzymes, reactive oxygen species, moonlighting proteins, metamorphic proteins, enzyme inhibitors, blocker drugs, amphotericin B, rapamycin, IAA94, HEDS assay

## Abstract

Chloride intracellular ion channel (CLIC) proteins exist as both soluble and integral membrane proteins, with CLIC1 capable of shifting between two distinct structural conformations. New evidence has emerged indicating that members of the CLIC family act as moonlighting proteins, referring to the ability of a single protein to carry out multiple functions. In addition to their ion channel activity, CLIC family members possess oxidoreductase enzymatic activity and share significant structural and sequence homology, along with varying overlaps in their tissue distribution and cellular localization. In this study, the 2-hydroxyethyl disulfide (HEDS) assay system was used to characterize kinetic properties, as well as the temperature and pH profiles of three CLIC protein family members (CLIC1, CLIC3, CLIC4). We also assessed the effects of the drugs rapamycin and amphotericin B, on the three CLIC proteins’ enzymatic activity in the HEDS assay. Our results demonstrate CLIC1 to be highly heat-sensitive, with optimal enzymatic activity observed at neutral pH7 and at a temperature of 37 °C, while CLIC3 had higher oxidoreductase activity in more acidic pH5 and was found to be relatively heat stable. CLIC4, like CLIC1, was temperature sensitive with optimal enzymatic activity observed at 37 °C; however, it showed optimal activity in more alkaline conditions of pH8. Our current study demonstrates individual differences in the enzymatic activity between the three CLIC proteins, suggesting each CLIC protein is likely regulated in discrete ways, involving changes in the subcellular milieu and microenvironment.

## 1. Introduction

**Figure 1:**
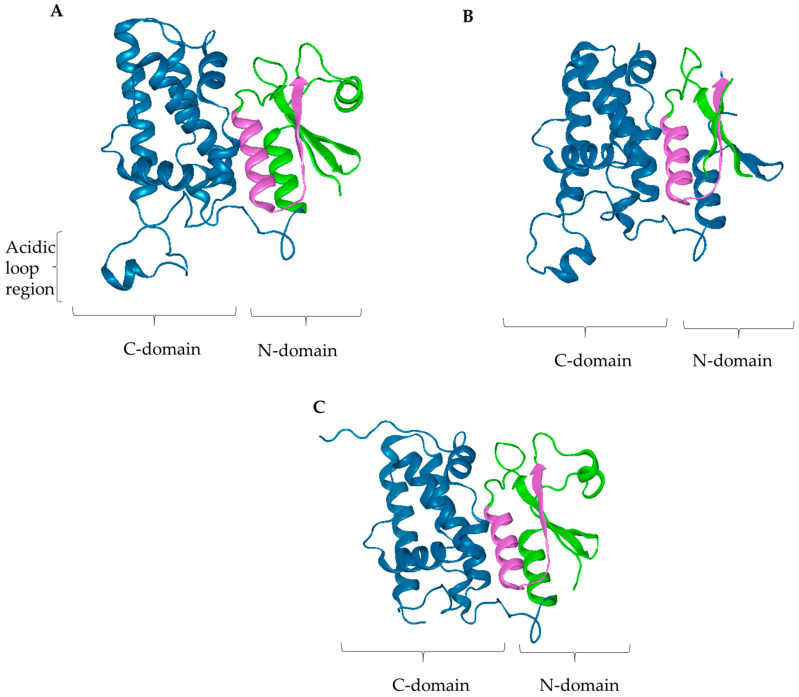
Three-dimensional structure of CLIC proteins. The C and N domain shown in blue and green, respectively, in each structure. (**A**) 3D ribbon structure of CLIC1 displaying the acidic loop region. The PDB code for this structure is 1K0M. (**B**) 3D structure of reduced CLIC3. The PDB code for this structure is 3FY7. (**C**) CLIC4 soluble structure. The PDB code for this structure is 2AHE. All figures were generated by using the DNASTAR Lasergene software v.15.3, Madison, WI, USA.

The human chloride intracellular channel (CLIC) proteins are a unique set of proteins with their peculiarity residing in their dual cellular localization; in fact, they are mostly found in a soluble monomeric form in the cytoplasm of resting cells, but they can also be found in a multimeric state as integral membrane proteins [1]. In addition, CLIC proteins do not follow the classical rule “one gene, one structure, one function”. Structural studies of proteins have typically been based on the presumption that proteins will adopt a single well-defined three-dimensional (3D) conformation within native conditions; however, an increasing number of proteins have demonstrated equivocal characteristics in their folding [2,3] (Figure 1). These non-classical proteins have been coined “metamorphic” as they have the potential to adopt more than one native 3D conformation, despite possessing the same amino acid sequence [2,3]. CLIC1 has been shown to adopt two distinct stable structural forms; therefore, it falls into this subset of non-classical metamorphic proteins [3]. These non-classical behaviors of CLIC proteins continue to intrigue, with the focus now turning towards their potential as moonlighting proteins [4,5]. 

Of the six human CLIC proteins, CLIC1 is one of the most extensively studied regarding its role in physiology and disease development. Apart from its ion channel activity, several other roles such as cell cycle regulation, apoptosis, modification of solute transportation, assisting adaptive immunity, and tissue homeostasis have been described [6,7,8,9,10,11]. Another significant aspect of CLIC proteins is their enzymatic activity, which further contributes to their functional versatility. CLICs demonstrate structural homology to the glutaredoxin (Grx) enzyme family, sharing a conserved glutaredoxin-like domain located in their N-terminal CLIC domain [12,13]. Additionally, CLIC proteins share a similar active thiol site motif (Cys-X-X-Cys/X) that either contains one highly reactive cysteine residue displaying a glutaredoxin monothiol motif as in the case of CLICs 1, 4, 5, and 6 or two cysteine residues forming a dithiol motif in the case of CLICs 2 and 3 [12,13]. This active site has been shown to couple with the peptide glutathione (GSH), which is used as a cofactor in the redox reactions catalyzed by members of the Grx family and now also by the CLIC protein family. We have previously shown that this monothiol or dithiol motif in CLICs undergoes reversible oxidation and reduction, enabling CLIC proteins to function as oxidoreductases capable of binding GSH in thiol-disulfide interchange reactions [12,14,15]. We have also shown that the monomeric conformation of CLIC1 displayed the highest levels of enzymatic activity compared to its dimer form, which was also greater compared to CLIC2 and CLIC4 when measured in 2-hydroxyethyl disulfide (HEDS) assays [12]. More recent research has shown that CLIC3 also acts as a glutathione-dependent oxidoreductase, targeting the reduction and regulation of transglutaminase2 (TGM2) and binding to cofactors [14]. More recent studies propose that CLIC4 and CLIC5 are involved in cardio protection from *in vivo* ischemia-reperfusion and, respectively, aid in maintaining calcium homeostasis and mitochondrial ROS generation [16,17]. CLIC5′s role in ROS generation further supports their status as “moonlighting proteins”, acting as both non-traditional ion channels that can also perform additional enzymatic activities; with their latter role further supported by their high structural homologies to the GSTs, GST-Ωs, and plant dehydroascorbate reductases (DHARs). 

While a wide array of biological functions has been ascribed to the CLIC proteins, drawing conclusions about the role of individual CLICs is difficult. This is due to the presence of six paralogues, with multiple CLICs co-expressed in most cells, leading to potential functional redundancy between family members. Therefore, exploring individual differences and specific enzymatic behavior under discrete environmental conditions, is crucial to untangling their individual physiological roles. Enzyme activity is well known to be influenced by temperature, pH, and substrate concentration [18,19,20,21]. Therefore, to gain deeper insights into the functioning of CLIC proteins, we set out to further characterize their oxidoreductase enzymatic activity.

## 2. Materials and Methods

The following reagents were all purchased from Sigma Aldrich: glutathione reductase (GR) from yeast, reduced glutathione (GSH), nicotinamide adenine dinucleotide phosphate (NADPH), HEDS, bovine plasma thrombin, kanamycin, isopropyl ß-D-1-thiogalactopyranoside (IPTG), tris(2-carboxyethyl) phosphine (TCEP), indanyloxyacetic acid (IAA-94), and amphotericin B solution. All other reagents used were of analytical grade. 

### 2.1. Site-Directed Mutagenesis, Protein Expression and Purification of Recombinant CLIC1, CLIC3, CLIC4, CLIC1- C24A, CLIC1- K37A, and CLIC1-C59A

The following annotations will be used to refer to each mutant, with each containing a single amino acid substitution to alanine: C24A, K37A, and C59A. The cDNA encoding the wild-type His-CLIC1 fusion protein (NP_001279), cloned into the pET-28a vector, was used to generate the point mutations using the QuikChange site-directed mutagenesis kit (Stratagen, La Jolla, CA, USA) according to the manufacturer’s instruction. CLIC1 cDNA, encoding the different mutations (C24A, K37A, and C59A) were then sequenced at Macrogen Inc. (Seoul, Republic of Korea) to confirm the incorporation of the correct mutations into the CLIC1-pET-28a plasmid. Following the conformation of the correct point mutation, each plasmid encoding a particular CLIC1 mutant was used to transform Escherichia coli (*E. coli*) BL21 (DE3) pLysS strains for overexpression of the recombinant CLIC1 wild-type and mutant proteins.

Glycerol stocks of *E.coli* BL21 (DE3) cells were transformed with the His-tagged PET28a (+) expression vector (Novagen) containing the coding sequence for either human CLIC1, CLIC3, CLIC4, or CLIC1 mutants (CLIC1-C24A, CLIC1-K37A, and CLIC1-C59A) as previously described [22]. The human CLIC4 was prepared in the pGEX4T-1 vector (AMRAD-Pharmacia) that coded for an N-terminal GST purification tag. The recombinant CLIC proteins (rCLIC) were grown in 2xYT medium containing kanamycin at a concentration of 30 µg/mL (Sigma Aldrich) or 100 µg/mL ampicillin in the case of CLIC4, and inducted with 1 mM IPTG (Sigma Aldrich) at 20 °C with overnight shaking at ~200 rpm. Cells were then harvested and the rCLIC proteins were purified as previously described [12] with the exception of GST-CLIC4 expressing cells which were resuspended in phosphate buffered saline containing 0.5 mM TCEP prior to sonication. All soluble cell lysates were collected after an additional centrifugation at 10,000× *g* for 40 min at 4 °C and subjected to affinity chromatography using either a Ni2+-NTA (Qiagen, Hilden, Germany) column for His-tagged proteins or a GSTrap 4B (GE Healthcare, Chicago, IL, USA) column for GST-CLIC4. The His-tag or the GST-tag was removed by an in-column thrombin enzymatic cleavage using an overnight incubation of bovine plasma thrombin (Sigma Aldrich, St. Louis, MO, USA) (30 NIH units per 1 L of bacterial culture) at 4 °C. The cleaved CLIC proteins were then collected in PBS buffer (10 mM phosphate buffer, 2.7 mM KCl, 140 mM NaCl, pH 7.4, and 0.5 mM TCEP) and further purified through size exclusion chromatography (SEC) (AKTA Pure/Amersham Pharmacia Biotech) using a HiPrep™ 16/60 Sephacryl^®^ S-100HR (Sigma Aldrich) or a HiLoad 16/600 Superdex 75pg (GE Healthcare) column and equilibrated in column sizing buffer (100 mM KCl, 1 mM NaN3, 20 mM HEPES pH 7.5, and 0.5 mM TCEP). The rCLIC proteins were verified by SDS-page (Figure S2) using a 4–15% Mini-PROTEAN TGX Stain-Free™ Protein Gels (BioRad) and for visualization of the protein bands, the separation gel was stained with Coomassie Brilliant Blue R 250 stain (Sigma Aldrich), and Western-blotting using their respective anti-CLIC antibodies (Santa Cruz, Santa Cruz, CA, USA). Recombinant protein concentrations were measured spectrophotometrically and calculated using the following extinction coefficient values of 0.647, 0.391 and 0.745 for CLIC1, CLIC3, and CLIC4, respectively, or by the Bradford protein assay (ThermoFisher Scientific, Waltham, MA, USA) according to the manufacturer’s instructions. The purified samples were then aliquoted and stored at −80 °C for future experiments.

### 2.2. HEDS Enzyme Assay

**Figure 2:**
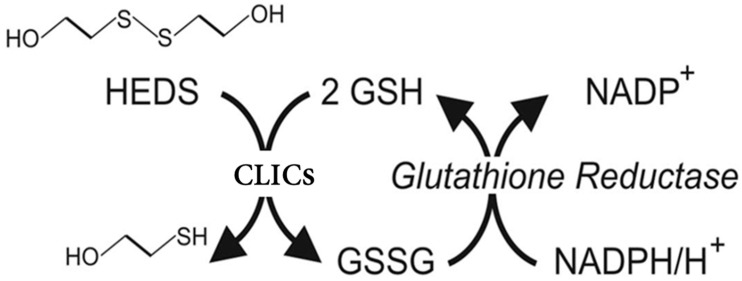
Mechanistic model represents HEDS assay. In this assay system, the CLICs protein acts as an enzyme by deglutathionylating the mixed disulfide between glutathione (GSH) and the beta-mercaptoethanol region of the HEDS substrate. Subsequently, the oxidized GSSG would be reduced again to GSH by the glutathione reductase, through catalyzing the consumption of NADPH.

HEDS assays act as a substrate for studying glutaredoxin enzymatic activity in high specificity and sensitivity. This assay was designed around the concept of protein de/glutathionylation, where the enzymatic activity of CLIC proteins assessed by its ability to catalyze the reduction of HEDS when combined with GSH and GR (Figure 2), by monitoring the consumption of NADPH, which reflects the formation of glutathione disulfide (GSSG), as NADPH is consumed by GR during the process [23]. 

Previous studies have demonstrated the oxidoreductase activity of the CLIC family member in the HEDS enzyme assay [12,15]. All HEDS enzyme assays were performed in a flat 96-well plate containing a final volume of 200 µL comprising of 10 µM final concentration of each protein added to a potassium phosphate buffer (5 mM/pH 7) that contained 1 mM EDTA, 250 µM NADPH, 1 mM HEDS, and 0.5 µg/mL GR. The mixture was incubated for 5 min at 37 °C, with the reaction initiated by the addition of 1 mM GSH. The consumption of NADPH was measured at A_340nm_ using the TECAN-Infinite M1000 microplate reader. Statistical analysis was performed using either one-way ANOVA with Dunnett’s multiple comparisons or the two-tailed student’s *t*-test and is presented as the mean ± SEM.

### 2.3. HEDS Enzyme Assay Conditions Use to Determine Enzyme Kinetics of CLIC1, CLIC3, and CLIC4

Enzyme kinetics of the CLIC1, CLIC3, and CLIC4 recombinant proteins were determined using varying concentrations of the HEDS substrate (0, 0.25, 0.5, 1, 2, 4, and 6 mM final concentration), wherein the activity of one unit of rCLICs was determined based on its capacity to oxidize 1 mmol of NADPH per minute, with a fixed concentration of 250 µM NADPH, 0.5 µg/mL GR, 1 mM GSH, and 10 μM fixed enzyme concentrations. Km and Vmax values were calculated from the Lineweaver–Burk plot.

### 2.4. HEDS Enzyme Assay Performed under Varying Temperature Conditions

In order to characterize and define the optimal catalytic activity of the purified CLIC1, CLIC3, and CLIC4 proteins, different biochemical conditions were applied using the standard HEDS enzyme assay procedure. This included pre-heating the protein samples across a range of temperatures, specifically: 0 °C, 30 °C, 37 °C, 42 °C, 50 °C, and 60 °C for 10 min. After that time, the residual activity of the CLIC proteins was assessed at 37 °C using the standard HEDS enzyme assay as previously described. To characterize the thermal effect on the enzymatic activity of recombinant CLIC proteins (CLIC1, CLIC3, and CLIC4), the recombinant proteins were subjected to a thirty-minute incubation at either 42 °C or room temperature prior to the HEDS assay being performed. Further thermal activity studies of CLIC1, CLIC3, and CLIC4 proteins were performed by running the HEDS assay at three different temperatures: 30 °C, 37 °C, and 42 °C in three individual experiments (*n* = 3).

### 2.5. Preparation of Transformed Bacterial Whole Cell Lysates with and without Heat-Shock

To investigate changes in oxidoreductase activity, an additional experiment was conducted using bacterial whole-cell lysates. The lysates of bacterial AF-CLIC1, AF-CLIC4, CLIC3, and pIRES2-EGFP were used at a final concentration of 10 μg. Following lysing bacterial cells, lysates were combined with a potassium phosphate buffer (5 mM, pH 7) containing 1 mM EDTA, 250 μM NADPH, 1 mM HEDS, and 0.5 μg/mL GR. Two sets of lysates were prepared: one was subjected to a heat-shock treatment at 42 °C for 30 min, while the other was kept at room temperature as a control. After the respective treatments, the lysate mixtures were incubated at 37 °C for 5 min. The reaction was initiated by adding 1 mM GSH. The consumption of NADPH, an indicator of the enzymatic activity, was measured at A_340nm_.

### 2.6. HEDS Enzyme Assay Performed under Varying pH Conditions

In order to determine the optimal pH for the catalytic activity of the rCLIC proteins and effects on the whole cell lysates enzymatic activity, the HEDS assay was run as previously described with the reaction mixtures prepared using buffers with a pH 5, 7, or 8.

### 2.7. HEDS Enzyme Assay Performed Using a Variety of Inhibitor Drugs

To evaluate the effects of drugs on the enzyme activity of CLIC1, CLIC3, and CLIC4, each protein was separately incubated at a final concentration of 10 μM with various drugs: IAA94, rapamycin (Selleck Chemicals AY-22989), and amphotericin B. The incubation took place for 1 h at 4 °C. Subsequently, the incubated CLIC proteins were mixed with 1 mM HEDS, 250 μM NADPH, 0.5 μg/mL glutathione reductase, and 5 mM potassium phosphate buffer (pH 7). This mixture was then incubated for 5 min at 37 °C, and the reaction was initiated by adding 1 mM GSH. Monitoring the NADPH consumption was done at A_340nm_.

## 3. Results and Discussion

### 3.1. Purified Recombinant CLIC Protein Enzymatic Activity Assessed via the HEDS Enzyme Assay

**Figure 3:**
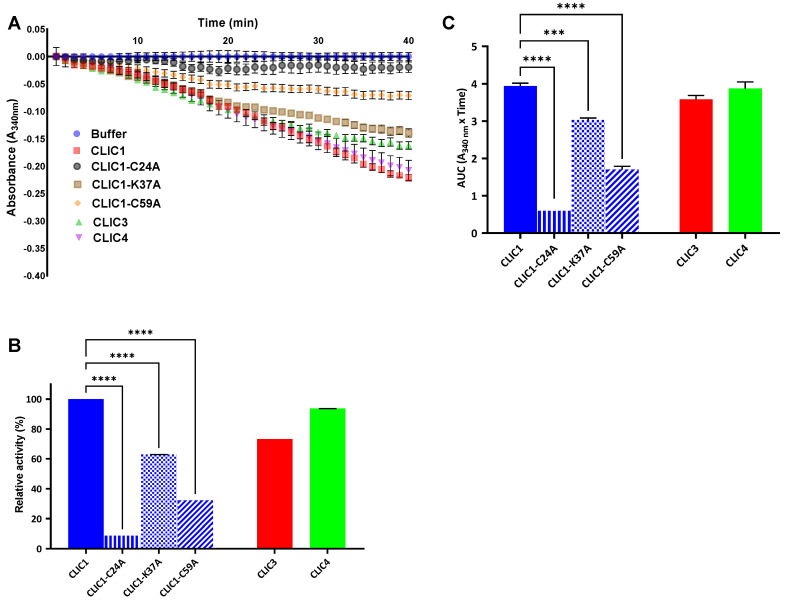
HEDS assay using CLIC1, CLIC3, CLIC4, and CLIC1 mutants. (**A**) The absorbance of NADPH was monitored over time at A_340nm_. (**B**) The relative activity of a final concentration of 10 µM of either CLIC1, CLIC3, CLIC4, or CLIC1 mutants was added to the HEDS assay, where the activity of the CLIC1 without mutation was defined as 100%. (**C**) Area under the curve of oxidoreductase activity shows that wildtype CLIC1 has the highest enzyme activity compared to its mutants. Results were analyzed with one-way ANOVA with Dunnett’s multiple comparisons test and expressed as mean ± SEM. *** *p* < 0.0005, and **** *p* < 0.0001, *n* = 3.

It has been demonstrated that CLIC1, CLIC3, and CLIC4 display glutaredoxin-like oxidoreductase enzymatic functions in their monomeric soluble state independent of their integral membrane ion channel activity [12,14,15]. As such, these recent discoveries of the enzymatic activity of CLIC family members necessitate further characterization of this activity. Structure–function studies have shown that Cys24 is located near the pore-forming region of CLIC1 (when in its membrane-bound form) and forms an active site monothiol motif in CLIC1 (CPFS) located in the common CLIC N-terminal domain [24]. This active cysteine and its equivalent found in all other CLICs is highly conserved. CLIC1′s Cys24 has also been shown to form a disulfide bond with Cys59 during oxidation that is critical in stabilizing its oxidized alternate structure. The mutation of Cys24 to alanine, causes a profound disruption of its enzymatic activity [12]. A similar mutation of Cys22 to alanine in the dithiol active site (CPSC) of CLIC3 also resulted in a significant loss of CLIC3 enzymatic activity [14]. To determine the functional activity of the purified CLIC1, CLIC3, and CLIC4 proteins, the HEDS enzyme assay was performed where buffer only (i.e., no CLIC proteins) and the CLIC1-C24A mutant were used as negative controls. As seen in Figure 3, the consumption of NADPH increases (resulting in a decreased absorption at A_340nm_) in the presence of CLIC1, CLIC3, and CLIC4, while this is greatly reduced in the case of CLIC1-C24A. This indicates that all three CLIC proteins reduced the HEDS substrate when coupled with GSH and GR in the presence of NADPH with CLIC3 showing the greatest activity and CLIC1-C24A mutant showing significantly reduced activity. 

HEDS assays were also conducted with CLIC1-C59A and CLIC1-K37A mutants, with both mutations showing a significant impact on the protein’s enzymatic activity. Cys59 in CLIC1, as described above, is involved in the stabilization of the oxidized form of the protein, through the formation of an intramolecular disulfide bond with Cys24 [24]. Its mutation caused a significant decrease in CLIC1 activity when tested in the HEDS assay, while the mutation of the charged residue Lys37 to alanine (K37A) also caused a significant reduction, albeit less pronounced, compared to Cys24A. Lys37 is located at the distal end of the first alpha-helix, which is also the predicted transmembrane domain of CLIC1. A previous study has shown this same mutation altered the biophysical properties of the CLIC1 ion channel activity in both artificial bilayers and cells [25].

### 3.2. Defining the Kinetic Parameters of CLIC Proteins’ Enzymatic Activity

**Figure 4:**
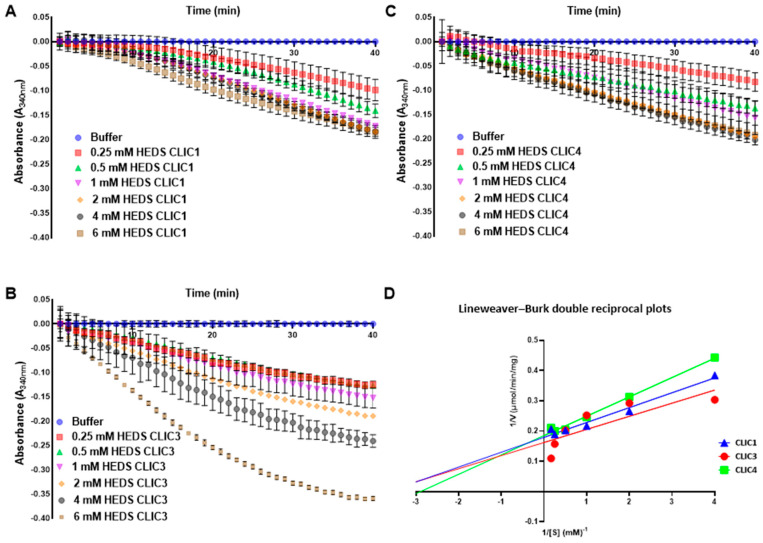
Representative graphs of CLIC1, CLIC3, and CLIC4 reduction of HEDS substrate and their different kinetic behaviors. A final concentration of 10 µM of either CLIC1 (**A**), CLIC3 (**B**), or CLIC4 (**C**) protein was added to the mixture of potassium phosphate buffer that contains 1 mM EDTA in pH 7, 0.5 ug/mL of GR, HEDS (0, 0.25, 0.5, 1, 2, 4, or 6 mM) and 250 µM of NADPH. After 5 min incubation at 37 °C, the reaction was initiated by 1 mM GSH addition. Subsequently, the absorbance of NADPH was monitored at A_340nm_. The error bars are representing of the standard deviation of three samples replicate. (**D**) Lineweaver–Burk double reciprocal plots of CLIC1, CLIC3, and CLIC4, Michaelis–Menten equation plot (Figure S3) mean ± SEM, *n* = 3.

To elucidate the kinetic profiles of each CLIC protein, we ran the HEDS assay using varying concentrations of the HEDS substrate (0–6 mM) while maintaining consistent concentrations of other reagents, as described in the methods section. Lineweaver–Burk double reciprocal plots were used to determine V_max_ and K_m_ and analyzed using non-linear regression (Figure 4). As presented in Figure 4, the reaction rates exhibited variations among CLIC1 (Figure 4A), CLIC3 (Figure 4B), and CLIC4 (Figure 4C) at specific HEDS concentrations, resulting in distinct V_max_ and K_m_ values. The kinetic parameters (K_m_, V_max_, and K_cat_) for the different CLIC proteins are presented in Figure 4D and summarized in Table 1. Specifically, the K_m_ values for CLIC1, CLIC3, and CLIC4 were 0.27, 0.26, and 0.34 mM, respectively. The K_m_ value of CLIC3 was lower than CLIC1 and CLIC4. A lower K_m_ value implies a greater affinity towards a substrate. Therefore, the results indicate that CLIC3, with the lowest K_m_ value (0.26 mM) has a higher affinity for the HEDS substrate. As a result, CLIC3 requires lower quantities of substrate to saturate its active site and efficiently reduce the disulfide bond. As a comparison, published kinetic studies of yeast dithiol Grx1 and Grx2 indicate these enzymes are able to reduce the mixed disulfide formed between GSH (1 mM) and the HEDS substrate, with an apparent K_m_ value of 0.12 mM and 0.7 mM, respectively [26]. A study of insulin reduction by thioredoxin has been reported to have a K_m_ value of 11 μM and a V_max_ of 4 μM·min^−1^ [27].

Additionally, CLIC3 showed the highest k_cat_/K_m_ values at the same enzyme concentration. Titration of HEDS substrate between (0–6 mM) demonstrated that the Vmax values CLIC1, CLIC3, and CLIC4 were 5.57, 6.15, and 5.39 μmol·min^−1^·mg^−1^, respectively, under the reaction conditions of 37 °C, pH 7.0. CLIC4′s lower V_max_ value of 5.396 μmol·min^−1^·mg^−1^ in comparison to that of CLIC1 and CLIC3 indicates a longer time is required for the enzyme to catalyze the chemical reaction. On the other hand, CLIC3 has the highest V_max_ value of 6.153 μmol·min^−1^·mg^−1^ indicating that the saturation of the enzyme active site occurs very quickly and hence, the chemical reaction is catalyzed faster in comparison to CLIC1 and CLIC4 (Figure 4). The k_cat_ indicates the maximum number of substrate molecules converted per second for a single catalytic site at a given enzyme concentration. The (S^−1^·mM^−1^) catalytic constant and physiological efficiency of CLIC1, CLIC3, and CLIC4 were 364.7, 434.3, and 271.5 1/s/mM, respectively. 

### 3.3. Evaluating Temperature Effects on Recombinant CLICs’ Enzymatic Activity 

We next studied the effects of temperature on the oxidoreductase activity of the purified CLIC proteins in the standard HEDS enzyme assay. We first looked at the effect of temperature on the protein’s stability by pre-heating the CLIC proteins prior to adding them to the assay, we then undertook studies looking at the effect of temperature on the enzymatic rate by running the HEDS assay at different temperatures. 

#### 3.3.1. Assessing Thermal Stability by Pre-Heating the Recombinant Proteins for 10 min across a Range of Temperatures

**Figure 5:**
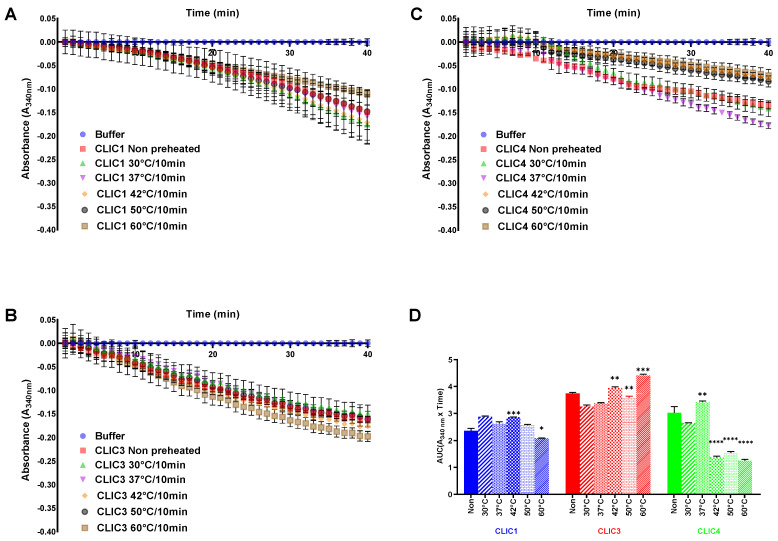
Summary graph of the effects of pre-heating on the catalytic activity of CLIC proteins. The catalytic profiles of pre-heating either CLIC1 (**A**), CLIC3 (**B**), or CLIC4 (**C**) across a temperature range of 0 °C, 30 °C, 37 °C, 42 °C, 50 °C, and 60 °C for 10 min before subjecting the proteins in the HEDS assay. (**D**) A comparison of the oxidoreductase activity of CLIC1 (blue), CLIC3 (red), and CLIC4 (green) following pre-heating at different temperatures prior to HEDS assay. Results were analyzed with one-way ANOVA with Dunnett’s multiple comparisons test and are expressed as mean ± SEM. * *p* < 0.05, ** *p* < 0.001, *** *p* < 0.0005, and **** *p* < 0.0001. *n* = 3.

Figure 5 summarizes the results following pre-heating of the CLIC proteins for only 10 min at different temperatures ranging from 30 °C to 60 °C immediately prior to their use in the HEDS assay and compared to a non-treated control (kept at room temperature, typically around 22 °C). In the case of CLIC4, pre-heating for 10 min above 37 °C resulted in a large reduction in its enzymatic activity, while showing highest activity when pre-heated to 37 °C (Figure 5C). CLIC1 only showed a significant reduction in activity at 60 °C, with maximal activity when pre-heated to 42 °C (Figure 5A). On the other hand, as seen in Figure 5B, CLIC3 showed less dramatic changes in its catalytic activity as a result of pre-heating. It is noteworthy that CLIC3 activity appears to increase when pre-heated to temperatures of 42 °C and 65 °C. Comparing the catalytic activities of CLIC1, CLIC3, and CLIC4 across different pre-heating temperatures, it is evident that CLIC3 shows greater heat resistance compared to CLIC1 or CLIC4.

#### 3.3.2. Assessing Thermal Tolerance of the Recombinant CLIC Proteins’ Enzymatic Activity by a Longer (30 min) (Pre) Heat Treatment

**Figure 6:**
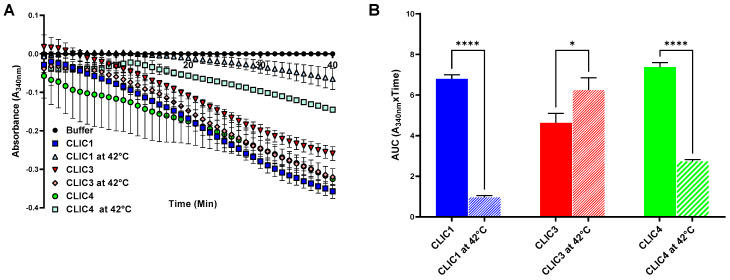
HEDS assay using recombinant CLIC proteins with or without heat. (**A**) The catalytic profiles of pre-heating either CLIC1, CLIC3, or CLIC4 at 42 °C for 30 min before subjecting the proteins in the HEDS assay. (**B**) A comparison of the oxidoreductase activity of CLIC1 (blue), CLIC3 (red), and CLIC4 (green) following pre-heating at 42 °C for a longer period of 30 min prior to HEDS assay. Results were analyzed with one-way ANOVA with Dunnett’s multiple comparisons test and are expressed as mean ± SEM. * *p* < 0.05 and **** *p* < 0.0001. *n* = 3.

To further compare the three CLIC proteins’ thermal tolerance, purified recombinant CLIC1, CLIC3, and CLIC4 proteins were subjected to heating at 42 °C for a longer period of 30 min immediately prior to their use in the HEDS assay and compared to a non-treated control. CLIC1 and CLIC4 were the most heat sensitive, both showing significant decrease in their oxidoreductase activity, post heating to 42 °C (Figure 6). On the other hand, CLIC3′s enzymatic activity tended to increase with the heat treatment, as previously described. Thus, we can conclude that CLIC1 and CLIC4 are heat sensitive proteins, while CLIC3′s enzymatic activity is maintained and perhaps enhanced at higher temperatures. 

**Figure 7:**
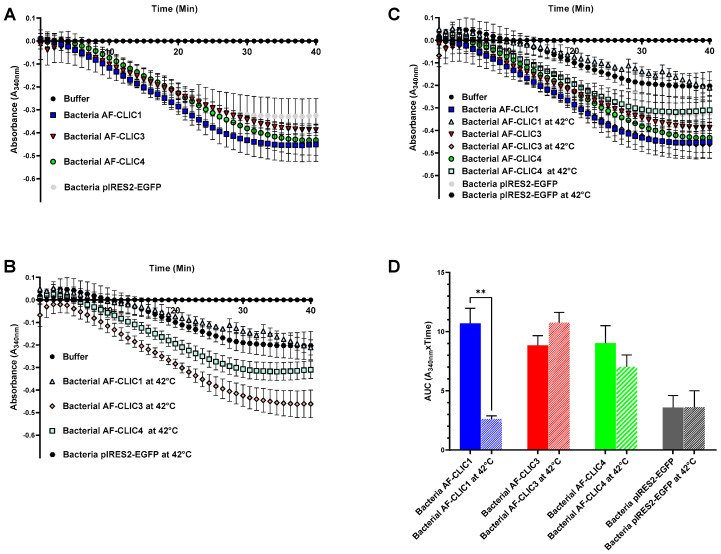
Comparing changes in the glutaredoxin activity of lysates from bacteria transformed with CLIC1, CLIC3, CLIC4, or empty control vector +/− heat shock. (**A**) XY plot showing that bacteria transformed with CLIC1, CLIC3, or CLIC4 have significantly higher levels of glutaredoxin-like oxidoreductase activity compared to the empty control vector. (**B**) XY plot showing that CLIC1 containing lysates were heat sensitive while CLIC3, CLIC4, and the empty control vector lysates were not significantly affected. (**C**) XY plot overlapping bacteria whole cell lysates with and without heat treatment. (**D**) Area under the curve shows that lysates from bacteria transformed with CLIC1 were significantly susceptible to heat shock while lysates of CLIC3 and CLIC4 transformed lines are more heat-stable. Results were analyzed with one-way ANOVA with Dunnett’s multiple comparisons test and are expressed as mean ± SEM. ** *p* < 0.001. *n* = 3.

A similar experiment was performed using cell lysates collected from bacteria expressing different recombinant CLIC proteins, namely CLIC1, CLIC3, or CLIC4, as well as a control lysate obtained from cells transformed with the empty pIRES2-EGFP vector (Figure 7). The lysates were subjected to heat treatment at 42 °C for a duration of 30 min prior to addition in the HEDS assay. The enzymatic activity of the lysates, as measured by the HEDS assay (Figure 7), showed a similar trend to the purified recombinant proteins (as seen in Figure 6). In Figure 7, lysates from CLIC1 transformed bacterial cells showed the greatest loss of enzymatic activity following heat treatment, compared to lysates of bacterial cells that were not heat treated or expressing CLIC3 or CLIC4. Similar to the purified recombinant proteins, heated CLIC3 lysates showed a tendency towards increased activity, while heated CLIC4 lysates again tended toward a lower activity. Interestingly, CLIC4 bacterial cell lysates exhibited greater heat tolerance compared to the purified CLIC4 protein. This is possibly due to the complex nature of the lysate which includes numerous components (including other CLIC-like proteins) and protein interactions, which may provide protection against heat effects that do not arise in the case of the recombinant purified protein. Control cell lysates transformed with an empty vector (both heated and non-heated), showed lower levels of oxidoreductase activity compared to CLIC-transformed cells, and this activity likely arises from native bacterial oxidoreductase enzymes or previously identified bacterial CLIC-like homologs [28].

### 3.4. Optimal Reaction Temperature for CLIC1, CLIC3, and CLIC4 in the HEDS Assay

**Figure 8:**
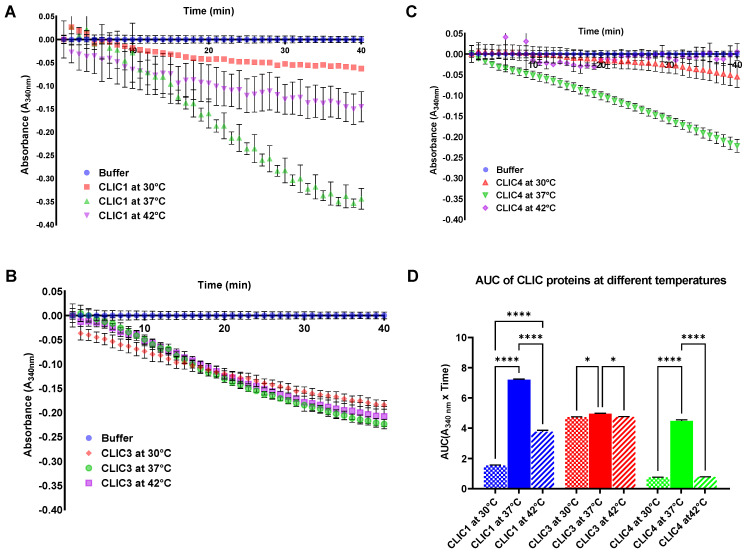
Summary graph of thermal activity of CLIC1, CLIC3, and CLIC4 proteins at 30 °C, 37 °C, and 42 °C. The catalytic profiles of assay temperature are either CLIC1 (**A**), CLIC3 (**B**), or CLIC4 (**C**). (**D**) A comparison of the oxidoreductase activity of CLIC1 (blue), CLIC3 (red), and CLIC4 (green) at 30 °C, 37 °C, and 42 °C assay temperatures. Results were analyzed with one-way ANOVA with Dunnett’s multiple comparisons test and are expressed as mean ± SEM. * *p* < 0.05, and **** *p* < 0.0001. *n* = 3.

Given the variation observed between the individual stability of CLIC proteins and their thermal tolerance, we next investigated the effects of temperature on the CLIC protein enzymatic activity, by performing the entirety of the HEDS assay at various temperatures. The HEDS experiment was done using purified recombinant CLIC1, CLIC3, and CLIC4 with the reactions run at 30 °C, 37 °C, or 42 °C, over a forty-minute period. Figure 8 shows that CLIC1 and CLIC4 oxidoreductase activity is optimal when the reaction is run at 37 °C, with significant activity reduction occurring at both 30 °C and 42 °C. CLIC3, however, showed little variation in its activity between the three different assay temperatures, again demonstrating its heat tolerance compared to CLIC1 and CLIC4 and its optimum of 37 °C. Overall, 37 °C appears to be an optimal temperature at which to run the HEDS assay for all three CLIC proteins studied, without any preheating and maintaining proteins on ice at 4 °C.

Similar studies of the Grx proteins found that the activity of *Chlorella sorokiniana* T-89 Grx enzyme was the most heat-stable Grx protein (activity retained at 80 °C for 30 min) [12,29], while an alternate study showed chlorella Grx has an optimal temperature at 37 °C [30]. A 2017 functional study of two CLIC homologs in the organism *Caenorhabditis elegans*, EXL-1 (excretory canal abnormal like-1) and EXC-4 (excretory canal abnormal ± 4), demonstrated EXL-1 translocates from the cytoplasm into the nucleus under heat stress [31]. The functional importance of this was supported by experiments using EXL-1 loss of function mutants, which demonstrated decreased heat resistance compared to wild-type animals, following exposure to heat stress (35 °C for 2 h) [31]. The authors of this study concluded that the CLIC homologs differ in their physiological functions, at least for the purposes of heat stress management [31]. Studies such as these, further support our proposal that the individual CLIC members likely have distinct activities, influenced by their surrounding environment conditions—temperature and pH—along with subcellular localization and cell type [6,7,8,9,10,11,32,33,34,35,36,37,38,39,40,41,42,43,44,45,46,47,48,49,50,51].

### 3.5. Evaluating Recombinant CLIC’s Enzymatic Activity under Varying pH Conditions

**Figure 9:**
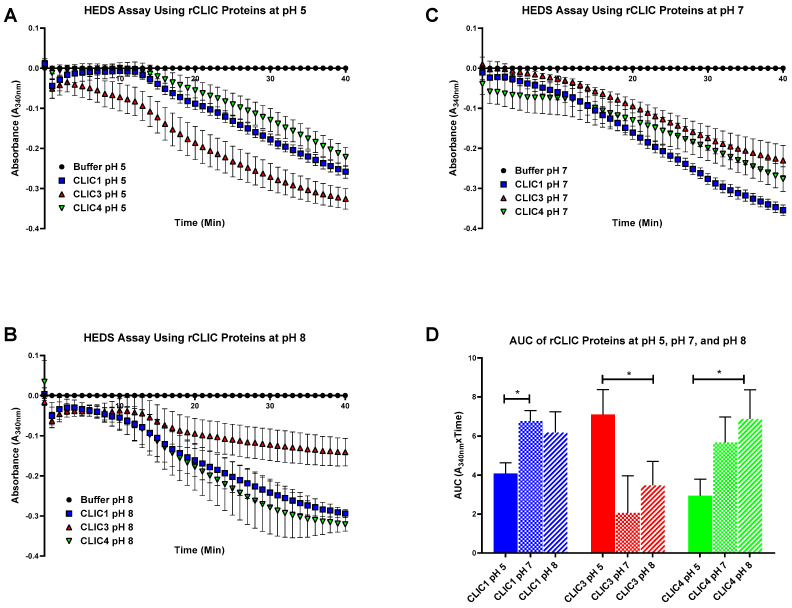
HEDS assay using recombinant CLIC Proteins at (**A**) pH 5, (**B**) pH 8, and (**C**) pH 7. (**D**) Area under the curve of recombinant CLIC Proteins at pH 5, 7, and 8. CLIC1 (shown in blue) shows an enzymatic optimum at pH 7. CLIC3 (green) prefers more acidic conditions such as pH 5 while CLIC4 (red) shows optimal activity at pH 8. Results were analyzed with one-way ANOVA with Dunnett’s multiple comparisons test and are expressed as mean ± SEM. * *p* < 0.05. *n* = 4.

pH is a critical parameter against which many cellular processes are exquisitely sensitive, including the actions of proteins and enzymes. Previously, studies of human CLIC1 ion channel activity have shown its activity increases under lower pH conditions [52,53], and a similar effect was found for the bacterial CLIC homolog, SspA [28]. Therefore, in order to determine the effects of pH on the CLIC proteins’ enzymatic activity, the HEDS assay was run using varying pH levels, specifically pH 5, 7, and 8. As seen in Figure 9, all three proteins demonstrated pH sensitivity. CLIC1 and CLIC4 were found to lose activity as the conditions became more acidic (pH 5), while they increased their enzymatic activity at more neutral pH values. On the other hand, CLIC3 was more enzymatically active at pH 5 and decreased its activity at pH 7 and pH 8. From our observations, it appears that the enzymatic functioning of CLIC1 and CLIC4 occurs optimally in more neutral to slightly alkaline conditions, while CLIC3 seems to perform optimally under more acidic conditions. For CLIC1 at least, its increased enzymatic activity at a more neutral pH is unlike its ion channel activity, which is greater under more acidic conditions, further highlighting the distinct moonlighting activity of these non-canonical enzyme proteins [52,53].

Numerous studies in the literature have shown that both protein processing and trafficking can be altered when luminal pH is changed, whether it be through post-translational modifications and processing, misdirection, or overall changes in the integrity of organelles [54,55,56]. Furthermore, several biological processes like protein–protein interactions [57,58], protein–ligand binding [59,60], protein interactions with membranes, [60,61,62] as well as peptide–membrane interactions [63], are also significantly influenced by changes in pH. Therefore, it was not surprising that studies of CLIC protein activity and localization have also demonstrated regulation by pH. Previous studies have shown that CLIC1 assembles in lipid bilayers in a pH-dependent manner [53], where a decrease in pH causes an increase in membrane interaction and insertion by CLIC1 [53]. It has also been suggested that when CLIC1 moves from the cytosol (pH 7) to a more acidic pH surface at the membrane (~pH 5.5), the lower pH ‘primes’ the structure of soluble CLIC, by lowering the activation energy barrier, which facilitates its conversion into a membrane-insertable form [64]. It is also likely that lower pH would also favor the maintenance of cysteine residues in a thiol (-SH) state. Therefore, similar structural changes may play a role in the reduced enzymatic activity of CLIC1 at acidic pH.

Generally, individual enzymes will demonstrate their own optimal catalytic activity at varying pH conditions. This term, optimum pH, is also dependent on the location within which the enzyme is found and normally functions within a specific cell or tissue. However, should the local pH rise or fall outside of this optimum, the enzyme’s structure may change, resulting in potential loss or enhancement of enzymatic activity. With CLIC1, CLIC3, and CLIC4, each was found to have a different optimal pH for their enzyme activity, suggesting that members of the CLIC family have distinct activities depending on cellular localization. Such a phenomenon was observed for the soybean vegetative lipoxygenases (VLXs). It was found that VLX A, B, and E showed optimal activity at pH 5.5, while VLX C and D had a pH optima between 6.5–7 [65]. Extrapolating from this, one could speculate that the six human CLIC proteins, likely function optimally within specific local cellular environments or distinct cellular sub-compartments, targeting specific substrates and distinct activities.

### 3.6. Assessing the Effect of Two Newly Identified CLIC4 Inhibitors (Rapamycin and Amphotericin B) on the CLIC Proteins’ Enzymatic Activity in the HEDS Assay

**Figure 10:**
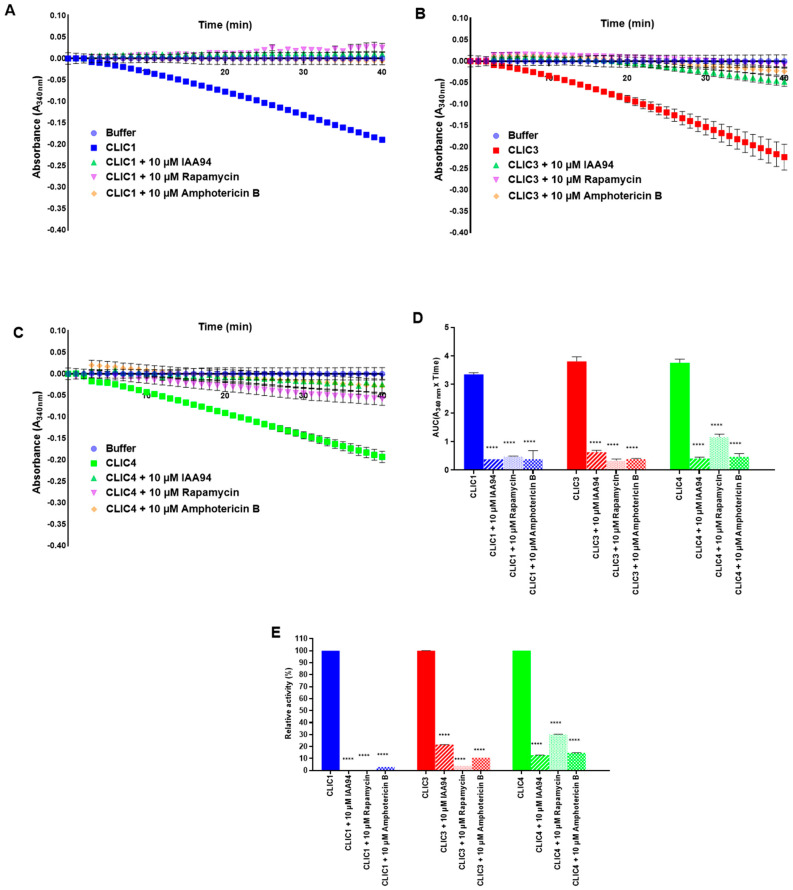
Effect of inhibitor drugs IAA94, rapamycin, and amphotericin B on the oxidoreductase activity of CLIC1, CLIC3, and CLIC4. The catalytic profiles from the HEDS assay for CLIC1 (**A**), CLIC3 (**B**), or CLIC4 (**C**). (**D**) A comparison of the oxidoreductase activity of CLIC1 (blue), CLIC3 (red), and CLIC4 (green) incubated with 10 μM final concentration of each inhibitor for 1 h. (**E**) The residual (%) enzymatic activity for each protein following inhibition with 10 µM final concentration of each inhibitor drug for 1 h, added to the HEDS assay. Activity of the CLIC1, CLIC3, or CLIC4 without inhibitors was defined as 100%. Results were analyzed with one-way ANOVA with Dunnett’s multiple comparisons test and are expressed as mean ± SEM. **** *p* < 0.0001; *n* = 9.

In a recent study utilizing a combination of computer-aided methods and experimental approaches, the drugs rapamycin and amphotericin B were identified as allosteric inhibitors of CLIC4, which also reversed stress-induced membrane translocation of CLIC4 and inhibited endothelial cell migration [66]. The diuretic drug IAA94 is a well-known ion channel blocker of chloride-selective ion channels, and can specifically inhibit the activity of CLIC channels when used at lower concentrations (≤100 µM) [67]. Furthermore, we have previously shown it can also inhibit CLIC enzymatic activity [12], and a cell study demonstrated the addition of IAA-94 to the growth media, caused cell cycle arrest in the G2/M phase, likely due to its blocking effect on the CLIC proteins [7]. In our current study, we tested the effect of the drugs rapamycin and amphotericin B on CLIC1, CLIC3, and CLIC4 enzymatic activity in the HEDS assay, with IAA94 included as a control. Both rapamycin and amphotericin B caused inhibition of the enzymatic activity of all three proteins, but with varying degrees of effectiveness. As depicted in Figure 10, both rapamycin and amphotericin B, like IAA94, caused significant inhibition of the enzymatic activity of all three proteins, with CLIC1′s activity virtually abolished by all three drugs. CLIC3 and CLIC4 showed varying levels of residual activity in the presence of either of the three drugs, ranging from 4.1–21.5% for CLIC3, and 12.8–30% for CLIC4, respectively. These findings confirm the previous study [66] and extend the findings to indicate that all three drugs inhibit the oxidoreductase catalytic activity of CLIC1, CLIC3, and CLIC4.

The highly conserved and ubiquitous expression of CLIC and CLIC-like proteins across vertebrates supports the hypothesis that these proteins play critically important roles within cells [1,7,12,13,24,68,69]. Initially, CLICs were shown to behave as atypical ion channels and have been ascribed a plethora of biological functions, ranging from ion channel activity to cell cycle regulation, and this is now extended to their enzymatic oxidoreductase activities [6,7,8,9,10,11,32,33,34,35,36,37,38,39,40,41,42,43,44,45,46,47,48,49,50,51,67]. 

## 4. Conclusions

CLIC proteins’ enzymatic activity is influenced by a variety of factors including temperature and pH. CLIC1 was observed to be highly heat-sensitive, with optimal enzymatic activity observed at neutral pH7 and at 37 °C, while CLIC3 had higher oxidoreductase activity at more acidic pH5 and was found to be heat stable under the conditions tested. CLIC4, like CLIC1, was temperature sensitive with optimal enzymatic activity observed at 37 °C; however, it showed optimal activity in more alkaline conditions of pH8. The current study also sought to define the kinetic profile of oxidoreductase catalytic activity of these CLIC proteins. Based on this characterization study, kinetic constants (V_max_, K_m_) for CLIC1, CLIC3, and CLIC4 were determined. By comparing these catalytic efficiencies, it was evident that these three CLIC proteins obey Michaelis–Menten and first-order kinetics. The V_max_ and K_m_ values for CLIC1, 3, and 4 proteins obtained using varying substrate concentrations showed that the CLICs have different affinity for the HEDS substrate at 37 °C, with the highest affinity shown by CLIC3 followed by CLIC1 and then CLIC4. Finally, the study also demonstrated that the drugs rapamycin and amphotericin B, like IAA94, cause significant inhibition of the oxidoreductase enzymatic activity of all three proteins, CLIC1, CLIC3, and CLIC4. 

## Figures and Tables

**Table 1:** Summary of the kinetic properties of CLIC1, CLIC3, and CLIC4.

	K_m_ (mM)	V_max_ (μmol·min^−1^·mg^−1^)	K_cat_ (1/S)	S^−1^·mM^−1^ (1/S/mM)
CLIC1	0.2738	5.573	99.87455	364.7719
CLIC3	0.2663	6.153	115.6579	434.3143
CLIC4	0.345	5.396	93.68056	271.5378

## Data Availability

Not applicable.

## Supplementary Materials

The following supporting information can be downloaded at: https://www.mdpi.com/article/10.3390/biom13091394/s1.
