# Supplementary material for: In Vitro Enzymatic Studies Reveal pH and Temperature Sensitive Properties of the CLIC Proteins
Source: Biomolecules. 2023 Sep 15;13(9):1394. doi: 10.3390/biom13091394 (PMC10526857; doi:10.3390/biom13091394)
Supplement: Supplementary file 1 [file biomolecules-13-01394-s001.zip › biomolecules-2610441-supplementary.pdf]

Supplementary Material

Figure S1 Comparing changes in Glutaredoxin activity when CLIC1, CLIC3 and CLIC4 are treated with 0.1% DMSO solvent.

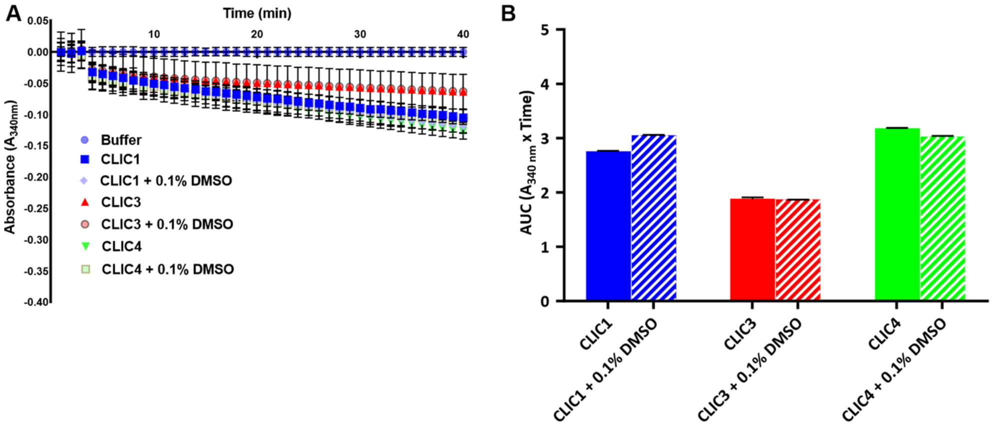

Figure S1. Comparing changes in Glutaredoxin activity when CLIC1, CLIC3 and CLIC4 are treated with 0.1% DMSO solvent. (A) An XY plot depicting the glutaredoxin-like oxidoreductase activity of CLIC1, CLIC3, and CLIC4 with and without 0.1% DMSO. (B) The area under the curve (AUC) demonstrates that there is no significant difference in the glutaredoxin activity of CLIC1, CLIC3 and CLIC4 treated with 0.1% DMSO compared to their untreated counterparts. Results were analyzed with one-way ANOVA with Dunnett's multiple comparisons test and are expressed as mean ± SEM. n=3.

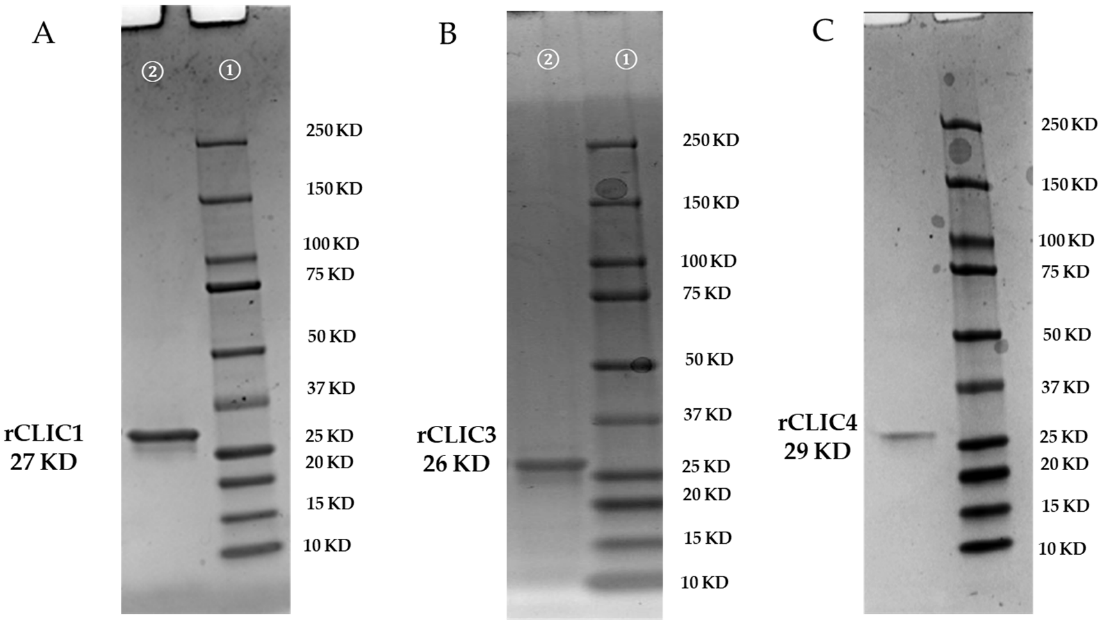

Figure S2. Polyacrylamide gel electrophoresis (SDS-PAGE) results of rCLIC proteins. A) rCLIC1, B) rCLIC3, C) rCLIC4.

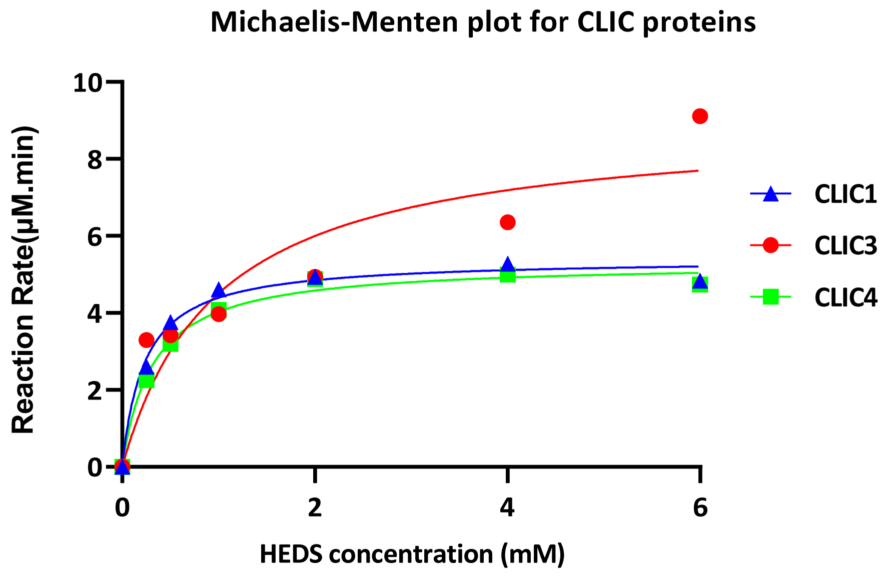

Figure S3: Michaelis-Menten plots of CLIC1, CLIC3 and CLIC4 representative graphs of CLIC1, CLIC3 and CLIC4 reduction of HEDS substrate and their different kinetic behaviours in Figure 2., mean ± SEM, n=3.

Table S1. Summary of the Kinetic Properties of CLIC1, CLIC3 and CLIC4 using Michaelis-Menten equation:

	Km (mM)	Vmax ($\mu mol \cdot min^{-1} \cdot mg^{-1}$)
CLIC1	5.408	0.2315
CLIC3	8.973	0.9916
CLIC4	5.307	0.3168
